# Sex-specific risk of cardiovascular disease and cognitive decline: pregnancy and menopause

**DOI:** 10.1186/2042-6410-4-6

**Published:** 2013-03-28

**Authors:** Virginia M Miller, Vesna D Garovic, Kejal Kantarci, Jill N Barnes, Muthuvel Jayachandran, Michelle M Mielke, Michael J Joyner, Lynne T Shuster, Walter A Rocca

**Affiliations:** 1 Departments of Surgery and Physiology and Biomedical Engineering, 200 1st St SW, Rochester, MN 55905, USA; 2 Division of Nephrology and Hypertension, 200 1st St SW, Rochester, MN 55905, USA; 3 Department of Radiology, 200 1st St SW, Rochester, MN 55905, USA; 4 Department of Anesthesiology, 200 1st St SW, Rochester, MN 55905, USA; 5 Department of Physiology and Biomedical Engineering, 200 1st St SW, Rochester, MN 55905, USA; 6 Department of Health Science Research, Division of Epidemiology, 200 1st St SW, Rochester, MN 55905, USA; 7 Department of Internal Medicine, Women’s Health Clinic, 200 1st St SW, Rochester, MN 55905, USA; 8 Department of Health Science Research, Division of Epidemiology, and Neurology, College of Medicine, Mayo Clinic, 200 1st St SW, Rochester, MN 55905, USA

**Keywords:** Brain imaging, Cerebral blood flow, Cognition, Estrogen, Hormone, Hypertension, Microvesicles, Preeclampsia, White matter hyperintensities

## Abstract

Understanding the biology of sex differences is integral to personalized medicine. Cardiovascular disease and cognitive decline are two related conditions, with distinct sex differences in morbidity and clinical manifestations, response to treatments, and mortality. Although mortality from all-cause cardiovascular diseases has declined in women over the past five years, due in part to increased educational campaigns regarding the recognition of symptoms and application of treatment guidelines, the mortality in women still exceeds that of men. The physiological basis for these differences requires further research, with particular attention to two physiological conditions which are unique to women and associated with hormonal changes: pregnancy and menopause. Both conditions have the potential to impact life-long cardiovascular risk, including cerebrovascular function and cognition in women. This review draws on epidemiological, translational, clinical, and basic science studies to assess the impact of hypertensive pregnancy disorders on cardiovascular disease and cognitive function later in life, and examines the effects of post-menopausal hormone treatments on cardiovascular risk and cognition in midlife women. We suggest that hypertensive pregnancy disorders and menopause activate vascular components, i.e., vascular endothelium and blood elements, including platelets and leukocytes, to release cell-membrane derived microvesicles that are potential mediators of changes in cerebral blood flow, and may ultimately affect cognition in women as they age. Research into specific sex differences for these disease processes with attention to an individual’s sex chromosomal complement and hormonal status is important and timely.

## Review

### Introduction

Sex differences from a medical perspective may include: 1) diseases/conditions specific to one sex, 2) diseases/conditions that disproportionately affect one sex, and 3) diseases/conditions having distinctly different causes, manifestations, outcomes (morbidity or mortality), or treatments depending on sex. In this context, sex is defined by the sex chromosomal complement and the presence of reproductive organs [1]. Cardiovascular disease and cognitive decline are two potentially related conditions which fall into the second and third categories. For example, the development of cardiovascular disease, including hypertension, occurs about 10 years earlier in men than in women, but it increases exponentially in women after menopause [2]. Conventional treatments for hypertension reduce blood pressure in both men and women, but these treatments are less likely to result in normotensive levels in women [3], suggesting that there are sex differences underlying these pathophysiologic processes [4-6].

Sex differences in autonomic function related to sympathetic control of the vascular resistance, and to the synthesis, uptake, and disposition of adrenergic neurotransmitters may explain the greater incidence of hypertension in men and the greater incidence of vasospastic diseases, such as migraine, Raynaud’s disease, and postural orthostatic tachycardia syndrome (POTS) in women [7]. In addition, sex differences in the composition of the vascular and cardiac extracellular matrix contribute to the greater incidence of diastolic heart failure (heart failure with preserved ejection fraction, HFpEF) and transient apical ballooning syndrome (Takotsubo cardiomyopathy) in women compared to men [8-10].

Cognitive health following a cerebrovascular event also shows sex differences. For example, post-stroke disability [11], stroke-associated cognitive impairment [12] and dementia [13] are greater in women than in men. By 2050, the prevalence of Alzheimer’s disease is estimated to reach 11-16 million in the United States [14,15]. The social and economic implications of this epidemic will be greatest in women because of their longer life expectancy and greater risk of dementia compared with men.

**Figure 1:**
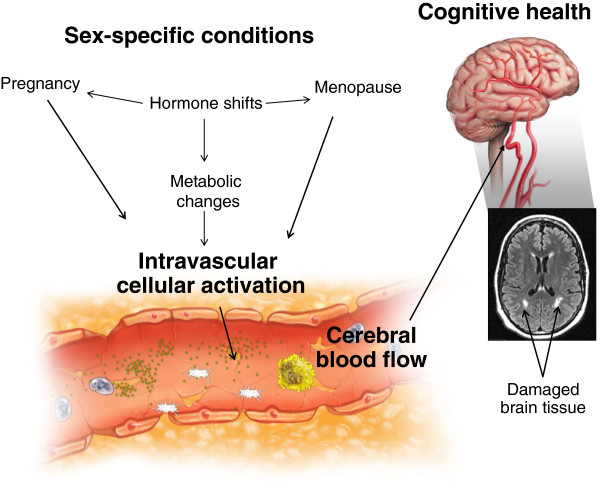
**Schematic of sex-specific conditions activating cells of the vascular compartment, including the vascular endothelium, platelets, leukocytes, and red blood cells, resulting in release/production of cell membrane-derived microvesicles.** Cell membrane-derived microvesicles are biologically active, themselves stimulating neighboring cells and releasing mitogenic, vasoactive, or inflammatory cytokines that ultimately affect vascular tone, including cerebral blood flow and brain function. Compromises in cerebral blood flow could negatively impact brain structure, and ultimately, cognition. The inset is a horizontal magnetic resonance image showing white matter hyperintensities (arrows) in the brain of a recently menopausal woman.

The physiological basis for these differences requires further research. Two conditions unique to women, pregnancy and menopause, which involve major hormonal changes, may contribute to distinct sex differences in morbidity, clinical manifestations, response to treatments, and mortality of cardiovascular disease and cognitive decline. This review examines the evidence suggesting that hypertensive pregnancy disorders, in particular, preeclampsia, affect cardiovascular risk in women as they age. In addition, it examines the evidence that menopausal hormone therapy (MHT) given close to the time of menopause reduces the risk for cardiovascular disease and cognitive decline. We will discuss the possible role of cell membrane-derived microvesicles in the blood that may affect endothelial function and sex-specific differences in the regulation of cerebral blood flow, as potential mechanisms mediating changes in cognition (Figure 1).

### Sex differences in cardiovascular pathophysiology

**Figure 2:**
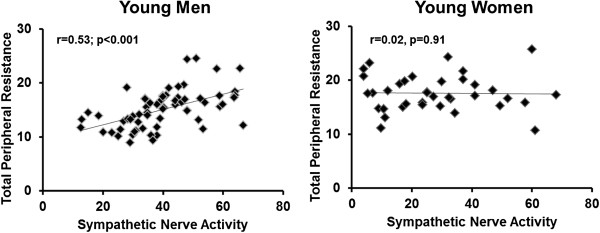
**The association between sympathetic nerve activity and total peripheral resistance in young men (n = 63; left panel) compared to young women (n = 37; right panel).** Data are combined from a series of studies investigating blood pressure regulation in healthy adults [33-36]. Each diamond represents an individual. Measurements of nerve activity were obtained using microneurography of the peroneal nerve under the same experimental conditions [37]. To control for fluctuations in sex hormones, women were studied only during the early follicular phase of the menstrual cycle.

Mechanisms involved in vascular and cardiac control and remodeling are regulated in part by sex steroid hormones. These mechanisms include the synthesis and degradation of norepinephrine [16,17], the expression of adrenergic receptors on vascular smooth muscle [18-22], the regulation of ion fluxes in cardiac and vascular smooth muscle [23-30], the production of endothelium-derived vasoactive factors [31,32] which affect total peripheral resistance (Figure 2, [33-37]), and cerebral blood flow [38-40]. Furthermore, regulation of extracellular collagen and elastin [41], and cellular apoptosis [42-46] may affect vascular and cardiac stiffness and remodeling processes that influence the development of vascular lesions and cardiac myopathies.

Thus, we suggest that two sex-specific conditions associated with major hormonal changes in women, specifically hypertensive pregnancy disorders and menopause, contribute to the development of cardiovascular disease, including hypertension and hypertension-related disorders, that impact brain structure and function.

#### Pregnancy-associated hypertension

**Figure 3:**
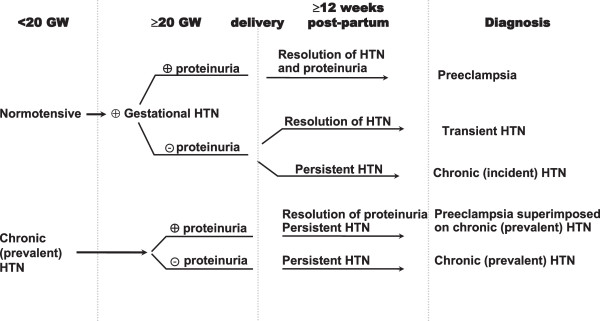
**Schematic of definitions, onsets, and consequences of hypertensive disorders of pregnancy.** GW = gestational week; HTN = hypertension.

Hypertensive pregnancy disorders cover a spectrum of conditions, including preeclampsia, gestational hypertension, chronic hypertension, and preeclampsia superimposed on chronic hypertension. Preeclampsia, unlike other hypertensive disorders of pregnancy, is associated with proteinuria (Figure 3) [47].

The National High Blood Pressure Education Program Working Group Report on High Blood Pressure in Pregnancy indicated that hypertensive disorders occur in 6% to 8% of pregnancies [47]. However, population-based studies evaluating the incidence of these disorders have not yet been conducted [48]. Consequently, available studies significantly differ in reporting their frequencies: 7% to 22% for hypertension in pregnancy, in general [49,50], and 1% to 8% for preeclampsia, in particular [49,51,52]. These differences result from lack of uniformity in defining the study populations and the clinical definitions of the disorders. In addition, the observed variations may have been further amplified by inaccuracies of diagnoses and differences in reporting chronic hypertension, which may predate pregnancy (chronic, prevalent hypertension), or occur for the first time during pregnancy and persist thereafter (chronic, incident hypertension) (Figure 3).

In addition to the short-term cardiovascular complications of preeclampsia (i.e., within three months postpartum), preeclampsia is associated with an increased risk of cardiovascular disease several years after the exposure. Two common study designs have been utilized to examine this long-term relationship. Case-control studies have examined women with cardiovascular events (e.g., myocardial infarction, venous thromboembolism, and stroke) and compared their pregnancy histories with those of event-free women of similar age (controls). These studies have suggested that, compared with women without cardiovascular events, women with cardiovascular events were more likely to have experienced a preeclamptic or hypertensive pregnancy disorder [53-56].

Registry-based cohort studies also suggest that hypertensive pregnancy disorders are associated with an increased risk of cardiovascular events [57-63] and mortality [60,63-67]. It is important to note that these studies have not fully adjusted for traditional cardiovascular risk factors. Without adjustment for these factors, it is not possible to determine whether the association between hypertensive pregnancy disorders and vascular outcomes is or is not related to traditional vascular risk factors (e.g., hypertension, family history, hyperlipidemia, smoking, and diabetes mellitus). Other limitations of the published studies include that they are often registry based (selected clinical series), have reported a limited number of outcomes (such as cardiovascular deaths), and have not assessed the impact of a hypertensive pregnancy disorder on age of onset of the cardiovascular event. This information may be clinically useful when individualizing risk profiles and intervention strategies for women with a hypertensive pregnancy disorder*.* Further, the diagnoses of preeclampsia and other hypertensive pregnancy disorders typically have been ascertained using codes from administrative data sources or self-reported events, rather than using accepted diagnostic criteria [58,61-63,68]. The four major studies that did confirm the diagnosis of preeclampsia using accepted clinical criteria included only mortality outcomes, and not the incidence or prevalence of cardiovascular events (cardiovascular morbidity) [64-67].

**Figure 4:**
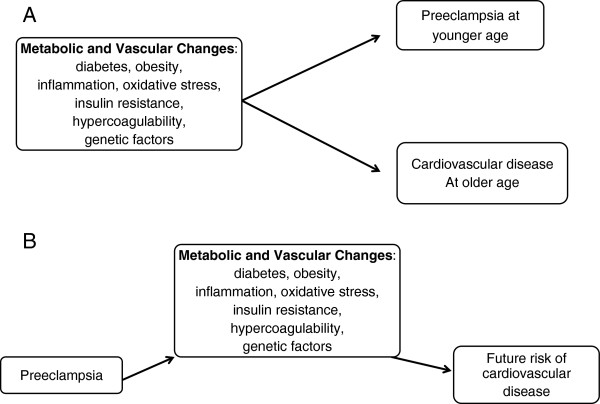
**Schematic representation of interactions among factors contributing to the development of preeclampsia and cardiovascular risk in women as they age.** Some risk factors, such as diabetes and obesity, may predispose women to hypertensive pregnancy disorders and preeclampsia at younger ages, and independently they may predispose women to cardiovascular complications and cognitive decline at older ages (**A**). Alternatively, preeclampsia itself might have direct causal effect (**B**) on vascular outcomes by inducing irreversible vascular and metabolic changes that may increase the overall risk for cardiovascular disease.

Major differences in the clinical presentations of preeclampsia and other hypertensive pregnancy disorders probably result from differences in their underlying pathophysiological mechanisms, which might have varying implications for cardiovascular disease later in life. However, the mechanisms underlying these associations are poorly understood. Some risk factors, such as diabetes and obesity, may predispose women to hypertensive pregnancy disorders and preeclampsia at younger ages, and independently they may predispose women to cardiovascular complications and cognitive decline at different times in a women’s life. In this situation, the pregnancy disorders have no causal relation to the later cardiovascular disease or cognitive decline. Alternatively, preeclampsia itself might induce irreversible vascular and metabolic changes that may increase the overall risk for cardiovascular disease (Figure 4). In this situation, the pregnancy disorders have a direct causal effect on vascular and cognitive outcomes.

In support of a direct causal effect, some studies showed that, despite normalization of blood pressure postpartum, these seemingly healthy women may demonstrate unfavorable metabolic and vascular changes [69], such as an impaired brachial artery flow-mediated (endothelium-dependent) dilatation, a measure of endothelial dysfunction, three years after the diagnosis of preeclampsia [70]. Also, micro-albuminuria, which may be a marker of endothelial dysfunction and/or renal injury, has been reported to be more prevalent following a preeclamptic pregnancy [71]. Echocardiographic studies showed an increased risk of concentric remodeling, eccentric hypertrophy, and impaired left ventricular relaxation one year postpartum in women with preeclamptic pregnancy compared with women with normotensive pregnancy [72].

Clarification of the mechanisms that underlie the association between hypertensive pregnancy disorders and future cardiovascular disease is important to establish more specific clinical guidelines for screening and/or treatment of cardiovascular disease in women. Current clinical guidelines recommend referral of women with a history of hypertensive pregnancy to primary care or cardiology in order to facilitate monitoring and control of cardiovascular risk factors, but there are no specific guidelines for management of these women [3].

#### Menopause

The risk of developing hypertension, ischemic heart disease, myocardial infarction and stroke increases in women after the onset of menopause, whether natural or surgically induced [2,73]. Estrogen-based treatments reduced the development of vascular lesions in experimental animals after oophorectomy [74-76]. Human studies have confirmed a reduced incidence of cardiovascular events and mortality in women using MHT for relief of menopausal symptoms after undergoing either surgical or natural menopause [73,77-85]. However, the timing of the initiation of such treatments is critical. Initiation of the treatment close to the time of menopause (i.e. within about 3 years) is more effective than delays in treatment of up to 5 years. This time period may represent a “window of opportunity” within which estrogenic treatments might be effective in reducing cardiovascular disease and associated events [86-88]. However, the impact of MHT on the development of hypertension at menopause remains controversial [89-92].

### Sex differences, hypertension, and cognitive aging

Compared with men, women are at increased risk for Alzheimer’s disease, the most common form of dementia [93-96], and their cognitive performance declines faster after the diagnosis of Alzheimer’s disease [97,98]. There also appears to be a sex-specific pharmacological effect of drugs targeting acetylcholinesterase activity [99]. Indeed, in experimental animals, sex differences have been found for nearly all cholinergic markers, including acetylcholinesterase activity, acetylcholine and acetylcholine receptor distribution [100-102]. These differences are likely related to sex hormones. Testosterone may interfere with the effects of cholinesterase inhibitors by decreasing the amount of drug that reaches the brain or by modifying the interaction of the cholinesterase inhibitor with cholinesterase [103,104]. However, reasons for these sex differences in the risk, progression, and treatment of dementia are not well understood.

Starting with Alois Alzheimer’s initial findings in the brain of a woman, changes in the microvessels have been repeatedly reported in the brain of patients with Alzheimer’s disease. These changes are now known to include cerebral amyloid angiopathy [105], endothelial degeneration [106], and vascular basement membrane alterations [107]. The notion that vascular factors are independent risk factors for Alzheimer’s disease was initially controversial. Vascular factors are the primary cause of vascular dementia, and one hypothesis was that such factors would only be associated with mixed cases of Alzheimer’s disease and vascular dementia. Additionally, it had been suggested that cardiovascular factors may be a consequence of Alzheimer’s disease, rather than a cause. However, in the early 1990’s, two publications reported an increased prevalence of senile plaques in patients with coronary artery disease [108,109], thus linking cardiovascular disease to Alzheimer’s disease. Since then, a number of epidemiological studies have confirmed that vascular-related conditions, such as hypertension [109,110], atherosclerosis [111], atrial fibrillation [112], diabetes [113,114], obesity [115], and stroke [116] increase the risk of Alzheimer’s disease. Vascular factors also affect the rate of progression after a diagnosis of Alzheimer’s disease [117]. Thus, vascular dementia and Alzheimer’s disease are no longer thought of as distinct entities, but as overlapping diseases.

It is possible that women with a history of hypertensive pregnancy disorders also have an increased risk of dementia through their increased risk for cardiovascular disease and Alzheimer’s disease later in life. This association is supported by the presence of white matter lesions, which appear on magnetic resonance imaging (MRI) as white matter hyperintensities (Figure 1) in women with severe forms of preeclampsia [118,119]. White matter hyperintensities are a recognized risk factor for both vascular dementia and Alzheimer’s disease [120,121]. Much remains to be learned regarding the factors contributing to their development, or to their causal relationship to changes in cognitive function.

However, no study has directly examined hypertensive pregnancy disorders as a risk factor for subsequent cognitive impairment. Two studies have suggested that preeclampsia and eclampsia are associated with self-reported worsening of cognitive function [122] and memory performance [123], but they did not systematically examine the association between hypertensive pregnancy disorders and domain-specific cognitive functioning later in life.

In women who develop eclampsia, a convulsive, severe form of hypertensive pregnancy disorder, the dilation of cerebral arteries is thought to result from a rapid increase in blood pressure, with resulting neurologic symptoms resembling those of a hypertensive encephalopathy [50]. With resolution of the hypertension, neurologic symptoms also resolve. However, the long-term consequences, for example, as women transition into menopause [49], on cerebral vascular function and residual effects on cognitive health remain unknown.

#### Cerebral blood flow and neuronal function

The brain does not have endogenous stores of energy. Therefore, brain metabolism depends on blood supplied by the cerebral circulation. In general, the dilatory capacity of the arterial vasculature, including that of the cerebral circulation, decreases with age [124-126]. This decrease is due, in part, to reduced production of endothelium-derived relaxing factors, such as nitric oxide, and increased production of endothelium-derived contracting factors, which may include cyclooxygenase products of arachidonic acid metabolism and superoxide radicals. These changes occur in the setting of decreased oxygen tension in the blood [38,124,127-129]. As sex-steroid hormones regulate many of these endothelium-derived relaxing and contracting factors [31,32], sex differences in the regulation of cerebral blood flow should be expected to manifest across the life span with changes in hormonal status.

**Figure 5:**
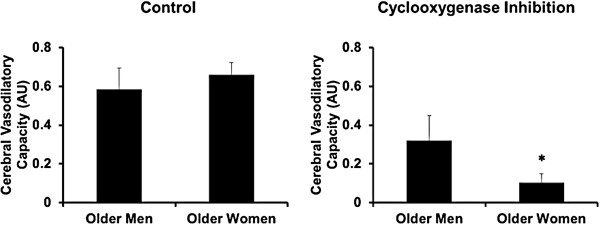
**Sex differences in cerebral vasodilatory capacity, after accounting for baseline cerebral blood flow velocity and mean arterial pressure, in men and women between 55-75 years of age (average 65 years; unpublished data provided by Jill Barnes, an author of this review).** The cerebrovascular responses to hypercapnia in age matched men (n = 6) and women (n = 6) are shown during control conditions (left panel) and during cyclooxygenase inhibition of vasodilating prostaglandins (right panel). Cyclooxygenase inhibition reduced the vasodilatory capacity (as area under the response curve, AU) only in older women (*p < 0.05). Data are mean ± SE.

One non-invasive method to measure vasodilator capacity of the cerebral arteries in humans is by transcranial Doppler during graded hypercapnia [130,131]. This technique has demonstrated that women have higher cerebral blood flow responses to hypercapnia compared with men of the same age, until the age of menopause [132]. However, this may be due, in part, to the higher baseline cerebral blood flow velocity in women of any age group. Although autoregulation should prevent changes in blood pressure from altering cerebral blood flow, emerging evidence suggests that sex differences in dynamic autoregulation exist [133]. Therefore, sex differences in “true” cerebral vasodilator capacity, when accounting for baseline flow velocity and acute changes in blood pressure, and their underlying mechanisms are unclear. Production of vasodilatory prostaglandins may be greater in women than in men, because the cyclooxygenase inhibitor indomethacin reduces the vasodilatory capacity to a greater extent in postmenopausal women than in age-matched men (Figure 5).

The vasodilatory capacity of the brachial artery decreases with preeclampsia [134] and menopause [135]. However, the effects of these conditions on the vasodilatory capacity of the cerebral circulation are unclear. For example, hypertensive pregnancy disorders, particularly preeclampsia, represent circumscribed events, and the future consequences of such events on cerebral vascular function have not been elucidated. In addition, although the risk of systemic hypertension increases at menopause, these effects of menopause on the cerebral circulation have not been defined. Furthermore, the effects of MHT on the cerebral circulation remain unclear [136-142].

Studies in experimental animals and cultured cells have consistently shown that estrogen enhances neurologic function and is neuroprotective, thus the maintenance of adequate estrogen levels could prevent or delay dementia in menopausal women. In observational studies comparing cognitive performance and dementia risk between a group of postmenopausal women who used MHT and a group of non-MHT users, MHT users performed better than non-users on the Modified Mini-Mental State Examination, and on tests of verbal fluency, verbal memory, and verbal and spatial working memory [143-148]. However, other observational studies did not identify a difference in cognitive function and dementia risk between the MHT users and non-users [148-151].

As with cardiovascular disease, controversy exists regarding whether MHT can preserve neurologic function and decrease the risk of dementia when administered early in menopause (onset of treatment within 3-5 years). In the Women’s Health Initiative Memory Study (WHIMS), dementia was not prevented in older women who initiated MHT later (after 5 years) into menopause [152,153]. However, several meta-analyses showed a 20% to 40% decrease in the risk of Alzheimer’s disease for women who use MHT early in menopause [154-157], in observational studies. Unfortunately, observational studies are subject to confounding effects. For example, better educated and healthy women are more likely to be MHT users and more likely to be compliant than are less-educated and less-healthy women (confounding by “healthy users” effect). Education and health are determinants of cognitive function by themselves, and these variables may not be fully adjusted during statistical analysis [152,158].

By contrast, randomized controlled clinical trials are not influenced by such confounding effects. Some randomized controlled trials have shown beneficial effects of MHT on cognition [159-161]. However, WHIMS, the largest randomized controlled trial designed to date to examine the effects of hormone therapy on cognitive function and incident dementia, found that conjugated estrogens, given to women at age 65 years and older (*late* into menopause), with or without medroxyprogesterone acetate, did not protect against dementia or cognitive decline. Rather, MHT substantially *increased* the risk of dementia and cognitive decline in these age groups [162-166].

It has been hypothesized that administration of estrogen during perimenopause, when endogenous estrogen concentrations are labile, protects against age-associated cognitive decline and dementia [167-173], but little is known about the mechanistic underpinnings of this hypothesis. In the rat hippocampus, aging leads to a loss of hippocampal estrogen receptor α, estradiol sensitivity, and loss of estradiol-mediated neuroprotection against global cerebral ischemia. However, estradiol administration to middle-aged rats was neuroprotective, supporting the hypothesis of a “window of opportunity” or a critical period for the initiation of MHT [174].

Other mechanisms by which estrogen might provide neuronal protection, as suggested from studies of animals and cultured cells include: 1) improving synapse formation on hippocampal dendritic spines [175-177]; 2) increasing the activity of choline acetyltransferase in the basal forebrain and hippocampus (choline acetyltransferase is a synthetic enzyme for acetylcholine, a neurotransmitter implicated in memory function, that is markedly reduced in Alzheimer’s disease) [178-181]; 3) reducing β-amyloid deposition in the brain and preventing the toxic effects of β-amyloid 1-42 on the neuronal mitochondria [45,182,183]; and 4) facilitating cerebral blood flow and acting as an antioxidant [40,184-186].

Following publication of clinical trial results from the WHIMS, there is a need for a randomized controlled trial to determine the neuroprotective effects of MHT in recently (< 3 years) postmenopausal women. However, determining these effects of MHT initiated close to menopause on the risk of dementia requires decades of follow-up, and is thus not feasible. A possible remedy to this obstacle is to use noninvasive imaging markers and measures of cerebral blood flow as short-term surrogate outcomes.

#### Surrogate imaging markers for investigating cognitive health

Volumetric MRI can be used to assess longitudinal effects of MHT on brain structure. Whole-brain and hippocampal volumes on MRI decrease during physiologic aging, accelerating after the fourth decade [187,188], with an annual rate of 0.2% decline in whole-brain volumes after age 54 years [189]. This decline in brain volume is consistent with autopsy studies showing that brain weight decreases after age 40 years. This decrease is thought to result from the degenerative processes of senescence such as cell shrinkage [187,188]. A direct relationship has been identified between hippocampal volumes on MRI and hippocampal neuronal density at autopsy in cognitively normal older adults and patients with Alzheimer’s disease [190]. Although, volumetric MRI is regarded as a surrogate for the structural integrity of the neurons in the elderly [191], similar studies of hippocampal volume in women close to menopause or with a history of hypertensive pregnancy disorders, and obtained in conjunction with assessments of cognition are needed.

A quantitative MRI marker of cerebrovascular health is white matter hyperintensities associated with small-vessel vascular disease in the brain [192]. Hypertensive renal disease is strongly associated with white matter hyperintensities [193], and better control of blood pressure slows their progression [194,195]. There is an association between white matter hyperintensity load and future risk of mild cognitive impairment [196-198]. On average, white matter hyperintensities are more common in patients with mild cognitive impairment and Alzheimer’s disease [199,200], in agreement with autopsy studies in which vascular disease was more common in patients with Alzheimer’s disease pathology [199,200]. Thus, quantitative analysis of the load of white matter hyperintensities may provide insight into the mechanisms by which menopause and hypertensive pregnancy disorders affect cognitive function in women.

Results of cross-sectional studies using MRI to assess the effects of MHT on brain morphology are mixed. One study found a decrease in gray matter volumes in MHT users compared to non-users [201], while another study found that MHT did not affect gray or white matter volumes [202]. Other studies found greater volumes of hippocampus [203-205], prefrontal cortex [206], cerebellum [203,207], temporal lobe gray matter [203,206], parietal lobe gray matter [203,206,207] and white matter [208] in cognitively normal MHT users compared to non-users. Some of these regions of brain morphology are involved in memory function.

Contrary to the findings from observational studies, data from WHIMS indicate greater hippocampal atrophy in postmenopausal women who are treated with hormones at age 65 years and older [209] and a slightly greater increase in white matter hyperintensities [210]. In WHIMS, women with low baseline cognitive function and high ischemic white matter hyperintensities were more prone to this MHT effect on the hippocampus, suggesting greater vulnerability of an already compromised brain to hormone treatment [209,210]. Furthermore, hippocampal volumes correlated with cognitive function in the treated group, suggesting MHT induces cognitive impairment through increased brain atrophy [163]. White matter hyperintensities in WHIMS were associated with baseline blood pressure, and a greater longitudinal increase in white matter hyperintensities occurred in those with higher blood pressure, demonstrating longitudinal blood pressure effects [211]. MRI findings in WHIMS are consistent with the previously reported decline in cognitive function and an increased risk of dementia with hormone treatment, and demonstrate that MRI-based measures of brain morphology are useful surrogates of cognitive function in postmenopausal women [210].

Diffusion tensor imaging is gaining acceptance as the preferred quantitative imaging technique for assessing white matter integrity in the aging brain. Data from experimental models suggest that the directionality of diffusion along the axonal projections measured with fractional anisotropy decreases with the loss of myelin and axons [212,213]. The reduction in fractional anisotropy in the white matter has been associated with the ischemic white matter hyperintensities in cognitively healthy elderly men and women. These fractional anisotropy reductions are not confined to hyperintense lesions but are also found in the normal appearing white matter [214,215]. One possible explanation for these diffusion abnormalities in the normal appearing white matter is that the decrease in fractional anisotropy may be antecedent to the white matter hyperintensities which are the end stage of ischemic vascular damage to the white matter [216]. The relationship between vascular risk factors and fractional anisotropy reduction in the white matter [215] further suggests that fractional anisotropy reduction in the aging brain may be a marker for subclinical cerebrovascular disease. Although the biological basis of diffusivity changes in the aging brain is yet unclear, the association between white matter fractional anisotropy and cognitive function underscores the potential of this new imaging technique [217-219].

Retention of the radio-labeled compound, Pittsburgh compound-B (PiB), monitored by positron emission tomography (PET) is a direct measure of the β-amyloid deposits in Alzheimer’s disease. A positive PET scan indicating the presence of β-amyloid deposits in cognitively normal adults is proposed as one of the research criteria for preclinical Alzheimer’s disease [220]. PiB binds to both β-amyloid 1-40 and β-amyloid 1-42 peptide species. Because β-amyloid 1-40 is the major β-amyloid peptide species within blood vessels, PiB is also sensitive to the β-amyloid associated vasculopathy or cerebral amyloid angiopathy [221]. Retention of PiB increases with age, and high PiB retention (at levels found in Alzheimer’s disease) was observed in 5.7% of normal individuals between the ages of 50 to 59 years, and in 19.0% of individuals between the ages of 60 to 69 years [222]. In the population-based Mayo Clinic Study of Aging, high PiB retention was present in 33% of cognitively normal older adults (average age, 79 years) [223]. Although estrogen is thought to modify the risk of Alzheimer’s disease, the effects of MHT on β-amyloid pathology need further investigation.

### Mediators of altered cerebral blood flow

Changes in cerebral blood flow may affect brain function acutely, as might occur with stroke or a preeclamptic event, or chronically, as might occur during changes in hormonal status (pregnancy and menopause) or during sustained hypertension [211]. To link these blood flow events to altered cognition, we can hypothesize that activation of some components in the blood (i.e. soluble components such as hormones or cytokines and/or cellular blood elements, including cell membrane-derived microvesicles), may reduce cerebral circulation, ultimately causing structural changes to the brain followed by cognitive impairment (Figure 1). Although this hypothesis requires rigorous testing, several lines of evidence point to its plausibility.

Blood platelets alter arterial diameter through their interactions with the vascular endothelium and smooth muscle cells [224-226]. These interactions are modulated by sex-steroid hormones [227-229]. Indeed, the content of several classes of vasoactive and mitogenic agents in platelets—including nitric oxide, prostacyclin, thromboxane A_2_, 5-hydroxytryptamine, tissue factor, tissue factor pathway inhibitor, transforming growth factor β, matrix metalloproteinases, and platelet-derived growth factors—varies with estrogen treatments [227,230-234].

Preeclampsia is characterized by a maternal hypercoagulable state, with increased intravascular coagulation and micro-thromboses that impair blood supply to several organs (Figure 4) [235-239]. Whether this hypercoagulable state or platelet activation contributes to overall cardiovascular risk or cerebrovascular vasodilatory capacity in women as they age remains to be determined.

Platelet activation may contribute to the progression of mild cognitive impairment or dementia. Significantly higher basal expressions of the platelet activation markers glycoprotein IIb/IIIa (PAC-1 binding) and P-selectin were observed in patients who developed cognitive decline at one year of follow-up (decrease of Mini-Mental State Examination score >4) compared with patients without decline (decrease in score ≤4) [240]. Furthermore, platelets from patients with Alzheimer’s disease and mild cognitive impairment contain higher concentrations of amyloid precursor protein and serotonin, and lesser amounts of epidermal growth factor and matrix metalloprotease-2 compared to healthy controls [241]. With ischemia, platelet aggregates accumulate both inside and outside of the blood-brain barrier and co-localize with toxic fragments of amyloid precursor protein. These observations suggest that progressive injury of brain parenchyma may be caused not only by degeneration of neurons destroyed during ischemia, but also by chronic damage to the blood-brain barrier, with the accumulation of amyloid precursor protein in the perivascular space, thereby leading to Alzheimer’s-disease pathology [242].

During cell-cell interactions, such as platelet interactions with other blood elements (i.e., leukocytes), cerebral vascular endothelium, or neurons, sealed membrane vesicles of <1 μm in diameter are shed into the circulation. Each microvesicle carries surface proteins/receptors characteristic of its cell of origin. Microvesicles are biochemically active and potentially important in several diseases, including cerebrovascular disease, preeclampsia, myeloproliferative disorders, and ischemic brain disease [243-247]. The composition of microvesicles and their numbers in the circulation depend on their cells of origin and the stimuli that trigger their production. Digital flow cytometry (FACSCanto^™^) and solid-phase fluorescence assays can be used to accurately identify and quantify the cellular origins of circulating microvesicles and their pathophysiologic characteristics [246,248,249]. Thus, it is possible to evaluate populations of circulating microvesicles, in early as well as late disease processes (e.g., development of white matter hyperintensities, β-amyloid pathology of Alzheimer’s disease, structural MRI changes associated with neuronal degeneration), and to study their associations with the cognitive health of women who have experienced preeclampsia, menopause, or who have used MHT. For example, in a subgroup of the women enrolled in the Kronos Early Estrogen Prevention Study (KEEPS) [250], increases in white matter hyperintensities over a four year period correlated with the number of activated, platelet-derived microvesicles at baseline [251].

These results suggest that blood borne microvesicles are part of a cascade of events that lead to the development of white matter hyperintensities. The effects of MHT on 1) the number and cellular origins of microvesicles, 2) the development of white matter hyperintensities, and 3) on direct measures of cerebral vasodilatory capacity remain to be determined. These studies can be extended to men in order to evaluate the association of testosterone deficiency with overall cardiovascular risk and cognitive decline.

## Conclusions

Viewing research and delivery of medical care through a “sex-based lens,” with attention to an individual’s sex chromosomal complement and hormonal status, is fundamental to individualized medicine. Changes in cerebrovascular function and cognitive health in women affected by female-specific conditions, such as preeclampsia and menopause, remain unexplored or controversial. Interdisciplinary research teams using population-based epidemiologic methods, structural imaging, and functional physiological and biochemical approaches are positioned to address these important and timely research questions. The ultimate goal is to improve preventive, diagnostic, and treatment strategies that could reduce sex disparities in disease and improve the health for women and men throughout their life spans.

## Abbreviations

MHT: Menopausal hormone therapy; MRI: Magnetic resonance imaging; WHIMS: Women’s health initiative memory study.

## Competing interests

None of the authors declare competing financial interests.

## Authors’ contribution

VMM, VDG, KK, JNB, MJ ,MMM, MJJ, LTS and WAR have contributed to the conception, drafting and editing of the manuscript. All authors read and approved the final manuscript.
